# Harnessing artificial intelligence for brain disease: advances in diagnosis, drug discovery, and closed-loop therapeutics

**DOI:** 10.3389/fneur.2025.1615523

**Published:** 2025-07-28

**Authors:** Su-jun Fang, Zhao-di Yin, Qi Cai, Li-fan Li, Peng-fei Zheng, Li-zhen Chen

**Affiliations:** ^1^Department of Pharmacy, The First Hospital of Putian City, Putian, China; ^2^College of Environmental and Biological Engineering, Putian University, Putian, China; ^3^Department of Neurology, Peking University People’s Hospital, Beijing, China; ^4^School of Medicine, Nankai University, Tianjin, China; ^5^School of Computer Science and Technology, University of Science and Technology of China, Hefei, China

**Keywords:** brain, artificial intelligence, drug discovery, personalized medicine, closed-loop system

## Abstract

Brain diseases pose a significant global health challenge due to their complexity and the limitations of traditional medical strategies. Recent advancements in artificial intelligence (AI), especially deep learning models like Convolutional Neural Networks (CNNs), Recurrent Neural Networks (RNNs), and Graph Neural Networks (GNNs), offer powerful new tools for analysis. These neural networks are effective at extracting complex patterns from high-dimensional data. By integrating diverse data sources-such as neuroimaging, multi-omics, and clinical information-multimodal AI provides the comprehensive view needed to understand intricate disease mechanisms. This review outlines how these technologies enhance precision drug development and enable closed-loop treatment systems for brain disorders. Key applications include improving diagnostic accuracy, identifying novel biomarkers, accelerating drug discovery through target identification and virtual screening, and predicting patient-specific treatment responses. These AI-driven methods have the potential to shift medicine from a one-size-fits-all model to a personalized approach, with diagnostics and therapies tailored to individual profiles. However, realizing this potential requires addressing significant challenges related to data access, model interpretability, clinical validation, and practical integration.

## 1 Introduction

The epidemic burden of brain diseases, encompassing conditions such as Alzheimer’s disease (AD), Parkinson’s disease, and various brain tumors, constitutes a pressing global health challenge. These diseases exhibit complex pathogenesis, with factors ranging from genetic predispositions and environmental influences to multifactorial interactions leading to neuronal degeneration and cognitive impairment (1, 2). The prevalence of such conditions continues to rise, prompting urgent calls for better diagnostics and therapeutics. Clinical advancements face substantial roadblocks, particularly in precision treatment and drug development. The blood–brain barrier (BBB) significantly complicates the delivery of therapeutic agents, leading to many drug candidates failing to penetrate effectively to their targets (3). Moreover, the focus on singular pathological mechanisms, such as amyloid-β in AD, has yielded limited success in drug approval (4). Central nervous system (CNS) drug candidates are significantly more likely than non-CNS therapies to fail in clinical development, reflecting late-stage attrition driven by inadequate brain exposure (5). Approval success rates for non-CNS indications are approximately 20%, whereas CNS therapeutics succeed at only 7–8% (6, 7). Furthermore, CNS pipelines incur extended development times-20% longer to develop and 38% longer to obtain approval compared to non-CNS programs (8). Innovations like tailored therapies and advanced biomarker systems are being explored, yet these solutions raise challenges of scalability and broad applicability in diverse patient populations (9).

The recent advancements in artificial intelligence (AI), particularly in neural learning networks and multimodal data integration, offer promising solutions for the challenges faced in diagnosing and treating brain diseases. Neural networks, such as convolutional neural networks (CNNs) and graph neural networks (GNNs), excel in automated feature extraction and complex pattern recognition, thereby facilitating rapid analysis of extensive and heterogeneous datasets from neuroimaging (MRI, PET) and molecular omics studies (10). Multimodal AI approaches enable the integration of diverse data sources-such as clinical records, multi-omics data, and real-time neuroimaging-enhancing the understanding of disease mechanisms that singular modalities might overlook (11). This comprehensive data analysis supports improved diagnostic accuracy, identification of novel biomarkers, and the acceleration of drug discovery by pinpointing promising therapeutic targets, ultimately paving the way for personalized medicine tailored to individual patients (1). Moreover, the application of AI technologies can help address the pressing need for timely and effective interventions in neurodegenerative diseases, significantly enhancing patient outcomes (12).

However, the widespread clinical adoption and practical implementation of AI-based approaches face considerable hurdles. Critical challenges include data standardization, privacy concerns, and the availability of diverse datasets, which complicate the training of robust AI models capable of generalization across various patient populations and clinical settings (13). Furthermore, issues surrounding model interpretability arise, as many AI systems operate as “black boxes,” limiting clinicians’ ability to understand and trust the decision-making processes behind their predictions (14, 15). Rigorous validation in real-world scenarios is essential to assess the reliability of these models in clinical practice, where patient variability can significantly impact outcomes (16, 17). While current discussions largely focus on AI’s reliability and explainability, addressing these limitations is vital to unlock the full potential of neural learning networks and multimodal AI in precision drug development and treatment systems for brain diseases (18). This mini-review aims to provide a synthesized overview of the precision drug development and closed-loop treatment system for brain diseases based on neural learning networks and multimodal AI.

## 2 Foundational technologies: neural learning networks and multimodal AI

**Figure 1:**
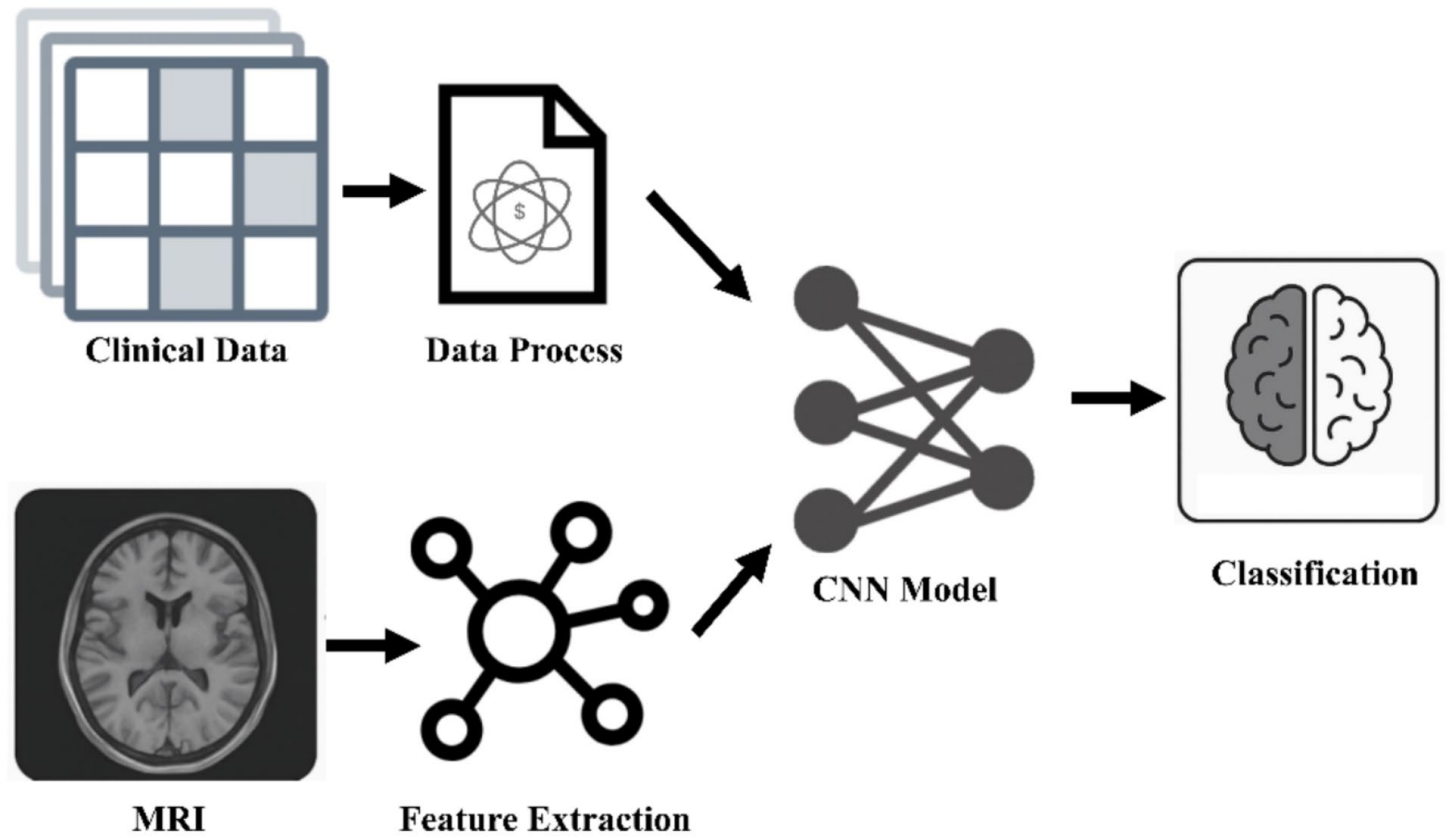
Multimodal medical analysis framework. The breakthrough of AI diagnosis system for neurodegenerative diseases lies in the integration of spatiotemporal MRI features and clinical trajectory data, and the recognition of non-static pathological patterns through convolutional neural networks, which significantly improves the accuracy of dynamic tracking. CNNs, convolutional neural networks.

The escalating burden of brain diseases necessitates a robust foundation for developing advanced computational strategies, particularly in precision drug development and closed-loop treatment systems. Central to this endeavor are neural learning networks and multimodal AI, technologies capable of processing complex, high-dimensional biomedical data that overwhelm traditional analytical methods (19). Neural learning networks, encompassing a range of architectures, provide the engine for extracting intricate patterns and relationships from this data, while multimodal AI enables the integration of information across diverse biological and clinical domains, offering a more comprehensive view of disease states than single data sources allow (20) (Figure 1).

Within the landscape of neural learning networks, several architectures have emerged as particularly relevant to brain disease research. CNNs, for instance, excel at processing grid-like data such as images and time-series signals (21, 22). In the context of brain diseases, CNNs are widely applied to neuroimaging data, including MRI and PET scans, for tasks such as lesion segmentation (23), brain atrophy analysis (24), and classification of disease states or subtypes (25, 26). They are also utilized for analyzing EEG/SEEG signals for seizure detection and prediction by capturing spatial and temporal features (27, 28). RNNs, notably Long Short-Term Memory (LSTM) networks, are designed to handle sequential data, making them suitable for analyzing time-series data like physiological signals (EEG/SEEG, vocal recordings) to identify temporal dependencies crucial for seizure prediction or tracking symptom progression (29, 30). GNNs are powerful for learning from data structured as graphs, which is highly applicable to biological networks such as protein–protein interactions (PPI) (31), drug-target interactions (DTI) (32), or relationships within knowledge graphs (KG) (33). GNNs can capture intricate relationships between entities, providing insights into underlying biological mechanisms and facilitating tasks like drug repurposing (32, 34). Transformer models, characterized by attention mechanisms, are increasingly used for their ability to weigh the importance of different data elements and capture long-range dependencies, finding application in sequence analysis and complex data integration (22, 35). Generative Adversarial Networks (GANs), while primarily known for generating synthetic data (36), are also employed in analysis tasks, for example, in denoising medical images or identifying complex patterns by learning underlying data distributions (24, 37). This diverse toolkit of neural learning architectures offers distinct advantages depending on the data structure and the specific task at hand. Sun R et al. developed a deep learning-based source imaging framework called DeepSIF, which significantly improved the spatiotemporal localization accuracy of drug-resistant focal epileptic seizure sources through synthetic training data and clinical verification (spatial specificity reached 96%, and spatial discreteness was only 3.80 ± 5.74 mm) (36).

The necessity for multimodal AI arises directly from the multifaceted nature of brain diseases. No single data modality provides a complete picture of the complex interplay of genetic, molecular, structural, functional, and behavioral factors that drive disease progression and affect treatment response (20, 38). Neuroimaging techniques, such as MRI and PET, offer crucial insights into structural and metabolic alterations in the brain, aiding diagnosis and tracking progression (26, 39). Omics data, including genomics, transcriptomics, and proteomics, shed light on the molecular underpinnings of disease, identifying relevant genes, pathways, and protein alterations (33, 38). Clinical data, ranging from patient history and symptom scales to physiological signals and behavioral assessments (vocal, gait), capture the phenotypic manifestations of disorders and patient responses to interventions (30, 40, 41). Molecular data, such as drug chemical structures and their interactions with biological targets, are fundamental for drug discovery and repurposing efforts (31, 32). Integrating these disparate data types through multimodal AI techniques is crucial. Various data fusion strategies are employed, from simple concatenation of features from different modalities to more sophisticated deep learning architectures designed for joint learning and integration (42, 43). Knowledge graphs (KGs) represent a powerful framework for organizing and integrating heterogeneous biomedical data by structuring entities (e.g., genes, drugs, diseases) and their relationships, facilitating knowledge discovery and inference that supports both diagnosis and drug development (32, 33). This comprehensive integration is seen as essential for moving toward precision medicine, where treatment decisions are informed by a holistic understanding of an individual’s disease based on multiple data layers (44).

While deep learning models demonstrate impressive performance metrics, the opacity of their decision-making processes can be a significant barrier to clinical trust and widespread adoption. Researchers are clearly grappling with how best to balance predictive power with explainability, exploring techniques like attention mechanisms or leveraging KGs not just for prediction, but for providing biological context to those predictions. Furthermore, the optimal strategy for integrating multimodal data appears highly dependent on the specific task and available data, indicating that a universal “best” approach remains elusive. Ensemble strategies, where multiple models are trained on distinct data modalities or architecture variants and their predictions are combined, offer a pragmatic route to both bolster performance and enhance interpretability in clinical settings. By aggregating outputs, ensembles not only mitigate individual model biases and stabilize predictions across heterogeneous inputs but also enable uncertainty quantification critical for clinical trust (45). Moreover, task-specific ensemble designs can flexibly accommodate varying data availability-ranging from multi-omics profiles to imaging-thereby sidestepping the search for a one-size-fits-all integration scheme and promoting robust, transparent decision support (46). Future advancements may lie not in finding a single dominant architecture or integration method, but in developing flexible frameworks that can be tailored and validated for specific brain diseases and clinical applications, potentially combining the strengths of different network types and fusion strategies to address the unique characteristics of each problem. The integration of neural learning networks and multimodal AI into brain disease research lays the groundwork for more accurate diagnosis, sophisticated prediction models, and accelerated drug development.

## 3 AI/ML for diagnosis and prediction of brain diseases

**Figure 2:**
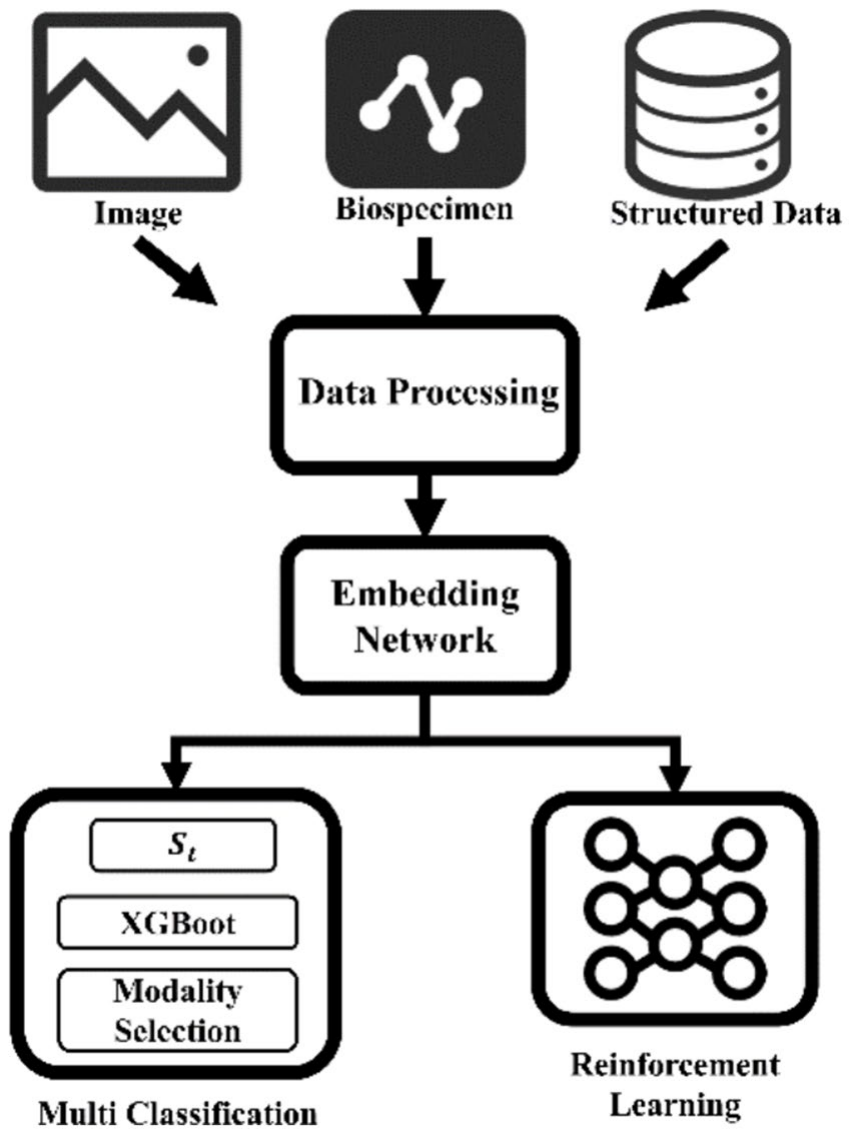
“Imaging-Biomics-Clinical” three-dimensional data fusion analysis architecture. Synergistically integrate imaging omics, multi-omics biomarkers and clinical time series data, build a cross-modal representation space through embedding networks, and innovatively couple XGBoost-driven interpretable classifiers and reinforcement learning decision modules. Not only can it achieve early detection of Tau protein deposition in Alzheimer’s disease, but it can also provide dynamic dose optimization strategies for individualized neuromodulatory therapies. MRI, Magnetic resonance imaging.

One prominent application area is the classification and subtyping of brain disorders. Distinguishing between different diseases or identifying specific subtypes within a heterogeneous condition like Parkinson’s disease or AD is critical for tailored treatment and improved outcomes (47, 48) (Figure 2).

Researchers have explored various strategies using neuroimaging data. For instance, deep learning models are applied to structural MRI (sMRI) or PET scans to classify patients into categories such as AD versus cognitively normal controls, or to differentiate between distinct stages or subtypes of cognitive impairment (25, 49). The theoretical basis often involves training the network to learn hierarchical features from the images that correlate with known pathological markers or clinical classifications (26). Multimodal imaging data, combining information from different MRI sequences (e.g., T1w, T2w, FLAIR) or integrating MRI and PET, is also leveraged to improve classification performance by providing complementary information (39, 50, 51). The problem of high-dimensional image data is often addressed through methods like feature extraction using pre-trained networks or dimensionality reduction techniques before classification (52). Similarly, in epilepsy, AI models are used to classify different types of seizures based on EEG or SEEG signals (27, 53). Beyond imaging, clinical data, including electronic health records (EHRs) and behavioral assessments, are also analyzed by AI/ML models for disease classification and subtyping, sometimes integrating linguistic information from clinical notes using large language models (LLMs) (40, 54). The comparison of different approaches in the literature highlights ongoing efforts to optimize network architectures and data fusion strategies for maximum diagnostic accuracy across various disease contexts (25).

The identification and discovery of biomarkers is another key area where AI/ML is making significant inroads. Biomarkers, whether imaging-based, molecular, or clinical, are essential for early detection, tracking disease progression, and providing targets for therapeutic intervention (21, 38). AI/ML models are used to identify imaging biomarkers such as patterns of brain atrophy in AD or Parkinson’s disease from MRI (55). Techniques like GANs can be used to synthesize normative brain images, and the difference between a patient’s scan and the synthesized norm can highlight individualized pathological changes, acting as a biomarker (24). Quantitative image analysis methods, often powered by deep learning for tasks like segmentation or feature extraction, are used to derive precise metrics from images that serve as biomarkers (52). For molecular biomarkers, AI/ML models, particularly those using KGs, analyze large-scale omics datasets to identify genes, proteins, or other molecular entities by predicting their association with disease risk or progression (33, 38). Clinical biomarkers, such as those derived from vocal or gait analysis, are also identified and analyzed by AI/ML models, often combining different types of clinical data to improve detection of conditions like Parkinson’s disease (30, 56). The theoretical basis for biomarker identification often relies on the AI model’s ability to learn features that strongly correlate with disease status or outcome, providing a data-driven approach to complement traditional hypothesis-driven research (57).

Predicting disease progression and patient prognosis is a crucial aspect of personalized medicine and clinical trial design. AI/ML models are developed to forecast the likelihood and timing of disease progression or to predict outcomes after interventions (e.g., seizure freedom after surgery, recovery from stroke) (58). Survival analysis methods, often combined with deep learning, are employed to predict time-to-event outcomes based on clinical, imaging, or molecular data (51, 59). For complex outcomes like seizure freedom after epilepsy surgery, AI models analyze multimodal data including neuroimaging and SEEG recordings to predict the success of surgical resection based on features related to the seizure onset zone (SOZ) and its connectivity. The prediction accuracy was improved by up to 40%, with the best algorithm achieving 96%/94%/96% prediction accuracy for core/expressive/receptive domain language improvement 2 months after surgery in an independent validation cohort (60, 61). Risk prediction models for conditions like stroke or atrial fibrillation recurrence are developed using AI/ML to analyze a variety of risk factors from clinical records and other data sources (62, 63). The problem of high-dimensional or imbalanced data in prediction tasks is addressed through techniques like feature selection algorithms or hybrid resampling techniques integrated into the AI framework (64, 65). Different modeling approaches, such as ensemble models or deep neural networks with attention mechanisms, are compared for their ability to improve prediction accuracy and provide interpretable insights into the factors driving outcomes (44).

There’s a notable diversity in the specific AI approaches and data combinations being explored, suggesting that no single “silver bullet” solution has yet been identified. One viewpoint emphasizes the power of deep learning to learn features directly from raw data, particularly imaging, arguing that complex patterns invisible to human eyes or traditional feature engineering methods can be captured effectively. An alternative perspective highlights the importance of incorporating prior biological knowledge, often through KGs or network-based approaches, believing that grounding the AI in known biology enhances interpretability and guides discovery. The integration of AI/ML into the diagnosis and prediction of brain diseases marks a significant step toward more precise and individualized patient care. By leveraging the power of neural learning networks and multimodal data analysis, researchers are developing tools capable of earlier and more accurate diagnosis, identification of critical biomarkers, and improved prediction of disease progression and treatment outcomes. The diversity of approaches, from deep learning on neuroimaging and omics data to network-based analysis and clinical prediction models, reflects the complexity of the diseases being studied and the active pursuit of optimal solutions.

## 4 AI/ML for precision drug development for brain diseases

A core approach in AI-driven drug discovery for brain diseases is the construction and analysis of KGs. Biomedical KGs integrate vast amounts of heterogeneous data from diverse sources, including genes, diseases, drugs, pathways, and clinical information, into a structured format that facilitates knowledge discovery and inference (32, 33). Projects like TarKG and AlzKB have specifically focused on building comprehensive KGs tailored for target discovery and drug repurposing in AD, integrating data from public databases, literature, and traditional medicine knowledge bases (66, 67). PharmKG provides a broad, multi-relational KG for various biomedical entities to support data mining tasks (68). The theoretical basis for using KGs lies in their ability to represent complex relationships that are difficult to capture with traditional matrix-based methods. Biomedical KGs, such as TarKG and AlzKB, are analyzed using GNNs to predict new drug-target interactions and identify candidates for repurposing (34, 69). This allows researchers to identify potential drug candidates and therapeutic targets by exploring the network structure and inferring connections based on existing knowledge (34). Some studies have highlighted the utility of incorporating additional information, such as pre-trained text embeddings from LLMs, to enhance the semantic richness of KG representations and improve link prediction performance (69). The insights gained from KG analysis can include identifying mechanisms of drug action, predicting adverse drug reactions, and suggesting novel drug candidates or targets (70).

Another crucial application is the identification of therapeutic targets. Brain diseases are often characterized by complex molecular mechanisms, and identifying the key proteins or pathways involved is essential for developing targeted therapies (33, 38). AI/ML models, can capture the interplay between different molecular layers, providing a more complete picture than analyzing each omics data type in isolation (71). Network-based approaches, often combined with deep learning, analyze the relationships between molecular entities (genes, proteins, metabolites) in disease-specific networks to prioritize potential targets (72). Examples from the literature include identifying targets for neurodegenerative diseases like DLK and JNK3 or exploring the roles of specific proteins like CD33 in AD (73–75). The theoretical basis here often involves identifying nodes or pathways within the network that are central or highly connected to disease-related entities, suggesting their importance as potential therapeutic targets (72).

Beyond target identification, AI/ML is transforming the process of molecular design and screening to find potential drug candidates. Virtual screening, using AI/ML models to predict the binding affinity of large libraries of compounds to a target protein, allows for rapid filtering of millions of potential drug candidates before costly experimental testing (76, 77). Deep learning models are trained on data of known DTIs, learning patterns in molecular structures that predict binding (78). These computational predictions are often complemented by molecular docking simulations, which model the physical interaction between a drug molecule and its target protein, providing structural insights into binding (76). Some studies integrate deep learning with molecular docking or dynamics simulations to improve the accuracy of binding prediction and identify promising candidates (31, 73). Generative models represent a more advanced application, using deep learning to design entirely new drug molecules with desired properties, such as targeting specific proteins or crossing the blood–brain barrier, rather than just screening existing libraries (79, 80). Predicting drug properties like BBB permeability is crucial for CNS drugs, and AI/ML models have shown high accuracy in this task (81). Evaluating potential toxicity and other ADMET properties early in the process using AI/ML helps to filter out problematic candidates, reducing later stage failures (35, 82). The theoretical basis for these molecular design and screening approaches often relies on AI’s ability to learn complex relationships between molecular structure and biological activity or properties. The computational predictions generated by these AI/ML methods are then validated experimentally using *in vitro* or *in vivo* assays to confirm their therapeutic potential (77). Case studies highlighting the discovery of specific compounds or the validation of predicted targets demonstrate the practical applicability of these AI-driven workflows (83, 84).

AlzKB is a publicly accessible knowledge graph integrating 118,902 entities and 1,309,527 relationships, including genes, proteins, compounds, and phenotypes, enable AI-driven drug repurposing for AD. By applying graph-based machine learning and link-prediction algorithms, investigators have identified novel therapeutic targets through similarity measures between AD and Parkinson’s disease subgraphs, highlighting candidate repurposed drugs for further validation (67).

Reflecting on the diverse landscape of AI/ML applications in brain disease drug development, the inherent difficulty of conventional drug discovery for these complex conditions has spurred a remarkable surge in creative computational approaches. It’s particularly striking to observe the interplay between data-driven discovery, relying on AI to find patterns in vast datasets, and knowledge-guided methods, which seek to embed existing biological understanding into the AI models. Is the most effective path one that lets the AI find completely novel associations, or one that uses AI to more efficiently search within a biologically plausible space? The literature suggests a complex answer, often highlighting the strengths of hybrid approaches that combine data-driven insights with biological constraints or leverage KGs to provide context to purely pattern-based findings. This tension is understandable; while pure data-driven models can uncover unexpected connections, their predictions may lack biological interpretability, hindering translation. Conversely, relying too heavily on existing knowledge might limit the discovery of truly novel therapies. The continuous cycle of computational prediction followed by experimental validation, as described in several studies, underscores the iterative nature of this process and the essential partnership between AI and traditional methods. Furthermore, the focus on drug repurposing, while practical, also raises questions about whether existing compound space is sufficient to address the fundamental complexities of these diseases, or if *de novo* molecular design powered by generative AI will be required to unlock truly transformative therapies. Both paths are being actively pursued, which reflects the multifaceted potential of AI in this crucial field as well as the pressing need for efficient treatments.

The integration of AI/ML tools for drug development is also increasingly focused on enabling precision medicine, tailoring therapies to individual patient profiles. Insights gained from diagnosis and prediction models, particularly those identifying disease subtypes or individual risk factors, can inform the drug discovery process (44). AI models can predict the efficacy of specific drugs for individual patients based on their unique clinical, genetic, or molecular profiles (85). This represents a significant progress from a one-size-fits-all approach to a more personalized strategy for therapeutic intervention. The ability to link identified targets, candidates, or predicted drug properties back to patient-specific data is a crucial step toward delivering on the promise of precision medicine for brain diseases (38).

## 5 AI/ML for closed-loop treatment systems and personalized medicine

One of the main objectives of brain disease research is to convert diagnostic and predictive insights into efficient therapeutic interventions and individualized patient care. Beyond merely identifying disease states, AI and ML are being used more and more to close this gap by directly influencing or even automating treatment plans. A crucial frontier is the shift to closed-loop systems and personalized medicine, which seeks to maximize results by customizing interventions to each patient’s distinct biological and clinical characteristics.

The use of AI/ML to forecast treatment response and suggest the best treatments is one important application. For conditions like epilepsy, recent studies trained CNNs to predict the likelihood of seizure freedom following surgery or the effectiveness of specific antiseizure medications based on patient data, including clinical history, neuroimaging, and even genetic information (86, 87). These models aim to assist clinicians in selecting the most appropriate treatment from the outset, potentially reducing the need for trial-and-error approaches and improving patient outcomes (86, 87). Similarly, in Parkinson’s disease, AI models are used to predict outcomes of deep brain stimulation (STN-DBS) based on multimodal data fusion, supporting personalized treatment planning (88). Predicting disease progression using AI/ML can also inform personalized recommendations, guiding medical practitioners on interventions to mitigate or postpone the effects of conditions like AD (59). The theoretical basis for these predictive models often involves learning complex, non-linear relationships between diverse patient features and treatment outcomes, leveraging the pattern recognition capabilities of deep neural networks and multimodal fusion techniques.

Enabling truly personalized treatment involves leveraging the wealth of data generated by AI/ML for a detailed understanding of an individual’s disease. Identifying specific molecular subtypes of Parkinson’s disease using multi-omics data analyzed by AI can guide the selection of therapeutic targets for drug repurposing (32). AI/ML models are also used to optimize drug development strategies specifically for neurodegenerative diseases by integrating drug-target information into models that predict potential therapeutic agents (89, 90). The concept extends to the design of drug delivery systems, where AI models can predict promising nanoparticle-drug combinations for neurodegenerative diseases, tailoring the delivery strategy to enhance efficacy and safety (91). Frameworks that integrate diagnosis, prediction, and drug recommendation within a unified AI architecture are being explored to provide a comprehensive approach to personalized management (21). Multimodal reinforcement learning is also being investigated to optimize medication recommendations in Parkinson’s disease based on diverse data modalities, aiming to assist clinicians in making informed decisions about tailored medication regimes (92). This emphasizes that personalization goes beyond simply predicting a general outcome; it involves understanding the individual’s specific disease manifestations and predicting how they will respond to different therapeutic options at a granular level (93).

The vision of closed-loop treatment systems represents the ultimate application of AI/ML in patient management, where AI goes beyond providing decision support to directly influencing therapeutic interventions in real-time. While still largely in the research phase for many brain diseases, the concept involves continuous monitoring of a patient’s state using physiological sensors (e.g., EEG, gait sensors), real-time analysis of this data by AI models to detect changes or predict events (e.g., seizures, “wearing off” periods), and automated adjustment of therapy (e.g., electrical stimulation, potentially medication delivery) based on the AI’s assessment (94–96). Adaptive deep brain stimulation (aDBS) in Parkinson’s disease serves as a prominent example, where AI analyzes neurophysiological biomarkers from implanted electrodes to adjust stimulation parameters dynamically (97). The theoretical basis involves training AI models to recognize specific physiological states or predict impending events from sensor data with high accuracy and low latency, enabling timely intervention (96, 97). Wearable sensors integrated with AI for real-time monitoring of symptoms like gait abnormalities or physiological states in Parkinson’s disease are being developed with the potential to inform automated or clinician-guided adjustments (94, 96). Similarly, real-time seizure detection from EEG using energy-efficient neural networks is explored for low-power wearable or implantable devices, which could form a component of a closed-loop system for epilepsy management (22, 41). The long-term goal is to create systems that can continuously monitor, analyze, and respond to a patient’s condition, providing precise, individualized therapy with minimal human oversight, particularly for conditions characterized by unpredictable fluctuations or acute events.

Adaptive deep brain stimulation (aDBS) exemplifies how closed-loop systems integrate AI/ML to tailor neuromodulatory therapy. These systems continuously monitor biomarkers, such as local field potentials and tremor metrics, to adapt stimulation parameters in real time, thereby reducing side effects and improving battery longevity (98, 99). In parallel, wearable EEG-based systems employ machine learning algorithms for ongoing electrophysiological monitoring and dynamic adjustment of therapeutic interventions. For instance, a study using a closed-loop wearable ultrasound DBS system based on EEG in mice provided promising data regarding seizure control, underscoring the potential for personalized treatments (100). Additionally, advances in integrated wearable EEG–fNIRS technology further support the development of responsive, patient-specific modalities that enhance clinical outcomes (101). A prominent clinical exemplar is Medtronic’s Percept™ PC implantable pulse generator, which utilizes BrainSense™ technology to chronically record neural signals and implement adaptive DBS algorithms in Parkinson’s disease and epilepsy, illustrating the translation of AI-driven closed-loop therapies into practice (8, 45).

However, real-world clinical adoption faces notable barriers such as latency, false positives, and battery life. Systems developed for seizure forecasting using weak self-supervised learning have reported high false positive rates, which not only risk unnecessary stimulations but also significantly impact the battery longevity of implanted devices (102). Battery constraints indeed become critical when frequent false alarms lead to excessive energy consumption, as illustrated in studies on absence seizure controllers (103) and efficient reservoir computing systems (104). Achieving ultra-low latency is essential for timely intervention; recent advancements in neuromorphic networks have achieved millisecond-scale detection (105), and wireless neuromodulation devices emphasize low-latency signal extraction (106). However, ensuring consistent performance in clinical environments remains a challenge. Addressing these issues through improved algorithmic precision and energy-efficient hardware is crucial for advancing personalized, closed-loop therapies.

While studies demonstrate AI’s ability to predict states like Parkinson’s “on” or “off” periods or impending seizures with high accuracy, the decision to automatically deliver a therapy based on these predictions involves a level of trust and accountability that current models, despite their performance, may not fully warrant. How do we ensure the safety and reliability of AI in triggering interventions, particularly when rare or unexpected events occur? How do we design systems that allow for human oversight and override when necessary? The debates implicitly present in the literature suggest that while the technical feasibility of closed-loop systems is advancing rapidly, the ethical frameworks, regulatory pathways, and clinical integration strategies required to ensure their safe and effective use in high-stakes brain disease management are still very much under development. Moving forward, it seems imperative that the conversation extends beyond algorithmic performance metrics to encompass the socio-technical aspects of deploying AI that interacts directly with human physiology, ensuring that these powerful tools augment, rather than replace, the essential human element of care.

By leveraging AI for treatment prediction, medication recommendation, and dynamic therapeutic adjustments, researchers are paving the way for more effective and individualized patient care (120). While promising systems like aDBS demonstrate the early potential of closed-loop approaches, challenges related to clinical validation, regulatory approval, and building trust in automated interventions remain.

## 6 Challenges and limitations

Despite the transformative potential of AI and ML for advancing diagnosis, prediction, drug development, and personalized treatment in brain diseases, significant challenges and limitations persist that temper the pace of translation into widespread clinical practice. These hurdles are multifaceted, spanning issues related to the data used to train and validate models, the models themselves, and the complex environment of clinical integration and regulatory oversight. Addressing these challenges is paramount for the responsible and effective deployment of AI in this high-stakes domain.

A primary set of challenges revolves around data. Brain diseases are inherently complex, and capturing their heterogeneity requires large, diverse datasets, which are often difficult to acquire and share due to ethical considerations, institutional policies, and the variability in data acquisition protocols across different centers (43, 64). The heterogeneity extends to the data modalities themselves; integrating disparate types like neuroimages, omics data, and clinical records, each with its own format, resolution, and inherent noise, presents considerable technical hurdles (20). Standardizing data collection and annotation is a labor-intensive process, and the quality of available data can be inconsistent, impacting model performance (52). Furthermore, many brain diseases, particularly rare conditions or specific subtypes, suffer from data scarcity and class imbalance, making it difficult to train robust models that perform well on underrepresented patient groups (30, 64). The need for real-time or near-real-time analysis in applications like seizure detection or closed-loop treatment systems further complicates data handling, requiring efficient pipelines for data acquisition and processing (41). Privacy and security are paramount concerns when dealing with sensitive patient data, necessitating robust data governance frameworks and potentially privacy-preserving techniques like federated learning to enable multi-site collaboration without centralizing raw data (107). The absence of standardized, large-scale, well-annotated multimodal datasets remains a significant impediment to developing and validating AI models that can generalize effectively across diverse patient populations and clinical settings.

Challenges related to the AI/ML models themselves are also considerable. One of the most frequently cited limitations is the lack of interpretability and explainability of complex deep learning models (56, 108). Often referred to as “black boxes,” these models can provide highly accurate predictions or classifications, but their internal decision-making processes are opaque (56). This lack of transparency poses a significant barrier to clinical trust and adoption, as clinicians need to understand why a model makes a particular recommendation or prediction to integrate it into patient care, especially for high-stakes decisions like surgical planning or drug prescription (108). While methods like attention mechanisms or leveraging KGs can enhance interpretability (41, 109), achieving full explainability that satisfies clinical requirements remains an active area of research (108). Generalizability is another critical challenge; models trained on data from one site or population often perform poorly when applied to data from different sources due to variations in data acquisition, patient demographics, or disease presentation (27). Developing robust models that can adapt to these variations or perform effectively on unseen data is essential for widespread clinical utility (41). Scalability and computational efficiency are also important considerations, particularly for real-time applications or analyzing massive datasets. While some models are designed for energy-efficient hardware (110), deploying complex multimodal AI models at scale can be computationally demanding.

Several studies have noted that models with high internal predictive accuracy (exceeding 80%) often perform poorly when externally validated, with accuracy sometimes dropping below 70% (111). For instance, an AI/ML-derived whole-genome predictor for glioblastoma experienced issues of overfitting and lack of generalizability, highlighting challenges in replicating performance in prospective trials (112). Similarly, organoid models used to simulate patient-specific responses can demonstrate considerable inter-patient variability, which current algorithms may not fully account for, contributing to subsequent trial failures (113). In neurodegenerative drug research, preclinical models continue to expose the risks of relying solely on internal metrics, as overfitting has been linked to high attrition rates in later-stage trials (114).

Algorithmic bias in precision drug development for brain diseases is a significant concern, as biased models have been shown to underperform by 15–20% for underrepresented populations, potentially leading to misestimated drug efficacy and safety (115). Federated learning emerges as a promising strategy by enabling decentralized model training across multiple institutions, thereby increasing dataset diversity and addressing privacy concerns that can exacerbate bias (115). Concurrently, explainable artificial intelligence (XAI) methods are pivotal for making complex machine learning (ML) decisions transparent, which facilitates the detection and mitigation of systemic biases affecting marginalized groups (115). The U. S. Food and Drug Administration’s AI/ML Software Action Plan further underscores the need for regulatory oversight, ensuring adaptive systems are validated on heterogeneous populations and maintain robust performance over time (116, 117).

The path to integrating AI/ML technologies into clinical practice and regulatory frameworks for brain diseases is fraught with its own set of challenges. Rigorous clinical validation is a non-negotiable step, requiring large-scale, prospective trials to demonstrate the real-world efficacy, safety, and clinical utility of AI-based tools (86, 118). The discrepancy sometimes observed between performance on internal validation datasets and external validation highlights the importance of testing models in diverse clinical settings. Regulatory bodies are still developing frameworks for evaluating and approving AI/ML-based medical devices, and the dynamic nature of some AI models that learn and adapt over time poses particular challenges for traditional approval processes (19, 118). Building clinical trust and ensuring clear accountability when AI is used to inform or directly influence patient care is also crucial (118). The acceptance of AI tools by clinicians depends not only on performance but also on usability, interpretability, and seamless integration into existing clinical workflows. Overcoming these barriers requires interdisciplinary collaboration between AI researchers, clinicians, data scientists, and regulatory experts to ensure that AI is developed and deployed responsibly and effectively.

Brain data often involves highly sensitive personal health information, such as neuroimaging scans, cognitive assessments, and even psychological states. Improper handling could cause significant ethical and privacy violations. Regulations like the EU’s General Data Protection Regulation (GDPR) and the US Health Insurance Portability and Accountability Act (HIPAA) establish frameworks for data use and protection (119). They require AI systems to embed compliance mechanisms-including data minimization, encrypted storage, audit trails, and informed consent processes-during design and application phases.

In brain disease diagnostics, the lack of transparency in black-box models limits physicians’ trust and adoption. This has driven widespread use of explainability techniques like SHAP, LIME, and Grad-CAM. Regulators increasingly require developers to clarify decision logic, particularly in high-risk scenarios. The US FDA and EU CE certification frameworks now oversee AI medical software, with FDA’s “Software as a Medical Device (SaMD)” guidelines and AI/ML lifecycle management recommendations outlining paths for registration, modification, and ongoing monitoring.

However, current certification processes primarily address static models. For AI systems with continuous learning capabilities, robust mechanisms for dynamic risk assessment and version tracking remain underdeveloped. These models self-optimize using new post-deployment data, risking “drift” that may invalidate original validation outcomes. Consequently, regulators are exploring “change protocol” mechanisms to log, review, and revalidate parameter updates.

The inherent complexity of brain diseases demands equally complex, data-intensive AI models, yet the very complexity that enables predictive power can undermine the interpretability, generalizability, and clinical trust essential for real-world impact. There is a visible struggle to balance the promise of cutting-edge performance with the need for models that are robust, fair, and understandable to both clinicians and patients. The varied attempts to address these issues-from developing novel model architectures to enhancing data fusion techniques or focusing on interpretability methods-reflect a field actively grappling with how to harness the power of AI responsibly. The disparities in reported performance across studies, even for seemingly similar tasks, underscore the sensitivity of these models to data characteristics and validation protocols. This variability is not a sign of failure, but rather a reflection of the field’s immaturity and the deep, complex problems it is trying to solve. The path forward requires a frank acknowledgment of these limitations and a concerted effort to build not just more powerful algorithms, but also the necessary infrastructure, standards, and collaborative frameworks that can support the rigorous development, validation, and responsible deployment of AI in brain disease research and clinical care.

## 7 Future directions

The journey toward fully realizing the potential of AI in brain disease research and clinical care is ongoing, with numerous promising avenues for future exploration. The reliance on large, high-quality, and diverse datasets for training robust AI models necessitates significant effort in standardizing data collection protocols and establishing secure, collaborative data-sharing platforms. Future directions will involve developing innovative approaches for federated learning and other privacy-preserving techniques that allow AI models to be trained on distributed datasets without compromising patient confidentiality. Furthermore, research will continue to explore methods for handling data heterogeneity, noise, and imbalance to ensure that AI models are robust and generalizable across diverse patient populations and clinical settings.

The development of truly closed-loop treatment systems represents a long-term goal that requires continued research and technological advancements. While current systems in areas like adaptive deep brain stimulation demonstrate early potential, future efforts will aim to create more sophisticated systems that can continuously monitor a wider range of physiological and behavioral signals, predict impending events with higher accuracy and lower latency, and automate therapeutic adjustments in a safe and effective manner. This involves developing energy-efficient AI models that can operate on wearable or implantable devices, as well as establishing robust feedback control mechanisms that can dynamically adjust interventions based on the AI’s real-time assessment of the patient’s state. Wearable biosensors for sleep and heart rate variability monitoring are emerging research frontiers for enabling real-time physiological feedback in closed-loop therapeutic systems. The integration of multi-omics data into these closed-loop systems could also provide more personalized and precise therapeutic adjustments, tailored to the individual’s unique biological profile.

The rapid evolution of AI technology itself will continue to drive future directions. Researchers will explore the application of novel AI architectures and techniques, such as advanced generative models for drug design or new forms of GNNs for analyzing complex biological networks, to address previously intractable problems in brain disease research. The potential of using AI to explore less-studied aspects of brain diseases, such as the interplay between different neurological conditions or the impact of environmental factors on disease risk, also represents a fertile ground for future research.

## 8 Conclusion

This review highlights the transformative potential of AI and ML in addressing complex brain diseases. By leveraging neural learning networks and multimodal AI, researchers can analyze diverse datasets from neuroimaging, genomics, and clinical records to uncover previously unattainable insights. Key advancements include improving diagnostic accuracy, discovering biomarkers for early detection, and accelerating precision drug development through methods like knowledge graph analysis and virtual molecular screening. AI is also paving the way for personalized medicine by predicting individual treatment responses and enabling adaptive closed-loop systems, such as adaptive deep brain stimulation. The core contribution of these technologies is their ability to decipher complex patterns and integrate heterogeneous data, guiding clinical decisions and drug discovery with enhanced precision.

However, significant challenges hinder widespread clinical adoption. Critical issues include data acquisition, standardization, and privacy. The “black box” nature of many deep learning models creates obstacles for clinical trust and regulatory approval, highlighting the need for more interpretable AI. Furthermore, ensuring models are generalizable across diverse populations, mitigating algorithmic bias, and conducting rigorous prospective clinical validation are essential. Future work must focus on creating robust and transparent AI, fostering collaboration to build high-quality multimodal datasets, and developing clear regulatory pathways. Overcoming these hurdles is crucial to fully realize AI’s potential to usher in a new era of precision neuroscience and improve care for patients with brain diseases.

## References

[1] Aguzzi A Kampmann M. Neurodegeneration enters the era of functional genomics. Science. (2023) 381:eadk5693. doi: 10.1126/science.adk5693, PMID: 37676963

[2] Hodson R. Alzheimer’s disease. Nature. (2018) 559:S1. doi: 10.1038/d41586-018-05717-6, PMID: 30046078

[3] Bender E. Getting Cancer drugs into the brain. Nature. (2018) 561:S46–7. doi: 10.1038/d41586-018-06707-4, PMID: 30258157

[4] Prillaman M. Alzheimer’s drug slows mental decline in trial - but is it a breakthrough? Nature. (2022) 610:15–6. doi: 10.1038/d41586-022-03081-0, PMID: 36175566

[5] Zhou X Smith QR Liu X. Brain penetrating peptides and peptide–drug conjugates to overcome the blood–brain barrier and target Cns diseases. Wiley Interdiscip Rev Nanomed Nanobiotechnol. (2021) 13:e1695. doi: 10.1002/wnan.1695 33470550

[6] Shi Z Chu Y Zhang Y Wang Y Wei DQ. Prediction of blood-brain barrier permeability of compounds by fusing resampling strategies and extreme gradient boosting. IEEE Access. (2021) 9:9557–66. doi: 10.1109/access.2020.3047852

[7] Luo X Ding Y Cao Y Liu Z Zhang W Zeng S. Few-shot meta-learning applied to whole brain activity maps improves systems neuropharmacology and drug discovery. iScience. (2024) 27:110875. doi: 10.1016/j.isci.2024.110875, PMID: 39319265 PMC11419810

[8] Holloway PM Willaime-Morawek S Siow R Barber M RnM O Sharma AD. Advances in microfluidic *in vitro* systems for neurological disease modeling. J Neurosci Res. (2021) 99:1276–307. doi: 10.1002/jnr.24794 33583054

[9] Servick K. Alzheimer’s experts greet China’s surprise approval of a drug for brain disease with Hope and caution. Science. (2019). doi: 10.1126/science.aba1117, PMID: 40328310

[10] Chang C-W Shao Q Mucke L. Tau: enabler of diverse brain disorders and target of rapidly evolving therapeutic strategies. Science. (2021) 371:eabb8255. doi: 10.1126/science.abb8255 33632820 PMC8118650

[11] Topol EJ. As artificial intelligence Goes multimodal, medical applications multiply. Science. (2023) 381:adk6139. doi: 10.1126/science.adk6139, PMID: 37708283

[12] Harris E. Aducanumab combined with focused brain ultrasound more effective. JAMA. (2024) 331:466–7. doi: 10.1001/jama.2023.27970, PMID: 38265836

[13] Zalewa K Olszak J Kapłan W Orłowska D Bartoszek L Kaus M. Application of artificial intelligence in radiological image analysis for pulmonary disease diagnosis: a review of current methods and challenges. J Educ Health Sport. (2025) 77:56893. doi: 10.12775/jehs.2025.77.56893

[14] Raghukumar R Nair A Raju A D‘Cruz A Joseph S. Ai used to predict Alzheimer’s disease. Int Res J Adv Engg MGT. (2024) 2:3647–51. doi: 10.47392/irjaem.2024.0541

[15] Jayaraj AF. The intersections of artificial intelligence, brain imaging tools and diagnostics for neurodegenerative diseases. J Stud Res. (2023) 12. doi: 10.47611/jsrhs.v12i3.5077

[16] Al-Kadi OS Al-Emaryeen R Al-Nahhas S Almallahi I Braik R Mahafza WS. Empowering brain cancer diagnosis: harnessing artificial intelligence for advanced imaging insights. Rev Neurosci. (2024) 35:399–419. doi: 10.1515/revneuro-2023-0115, PMID: 38291768

[17] Ahmed H Mo D Samaila BB. Current challenges of the state-of-the-art of AI techniques for diagnosing brain tumor. Mater Sci Eng Int J. (2023) 7:196–208. doi: 10.15406/mseij.2023.07.00224

[18] Ratnakar A Sawant S Karajagikar J. Explainable AI-driven deep learning for neurological disease diagnosis using MRI: a systematic review and future directions. Int J Sci Res Arch. (2025) 14:1799–832. doi: 10.30574/ijsra.2025.14.2.0533

[19] Suarez JI. Big data/Ai in Neurocritical care: maybe/summary. Neurocrit Care. (2022) 37:166–9. doi: 10.1007/s12028-021-01422-x, PMID: 34966957

[20] Liu XF Lu ZJ. Progress of bioinformatics studies for multi-omics and multi- modal data in complex diseases. Chin Sci Bull-Chin. (2024) 69:4432–46. doi: 10.1360/tb-2024-0416

[21] Wang YP Yang YF Li S Su ZC Guo JJ Wei PH. Automatic localization of seizure onset zone based on multi-epileptogenic biomarkers analysis of single-contact from Interictal Seeg. Bioengineering-Basel. (2022) 9:20. doi: 10.3390/bioengineering9120769, PMID: 36550975 PMC9774098

[22] Peng PZ Xie LP Wei HK. A deep Fourier neural network for seizure prediction using convolutional neural network and ratios of spectral power. Int J Neural Syst. (2021) 31:2150022. doi: 10.1142/s0129065721500222, PMID: 33970057

[23] Aminpour A Ebrahimi M Widjaja E. Lesion segmentation in paediatric epilepsy utilizing deep learning approaches. Adv Artif Intell Mach Learn. (2022) 2:422–40. doi: 10.54364/AAIML.2022.1128

[24] Shi R Sheng C Jin SC Zhang Q Zhang SY Zhang L. Generative adversarial network constrained multiple loss autoencoder: a deep learning-based individual atrophy detection for Alzheimer's disease and mild cognitive impairment. Hum Brain Mapp. (2023) 44:1129–46. doi: 10.1002/hbm.26146, PMID: 36394351 PMC9875916

[25] Chen YS Wang LZ Ding BJ Shi JS Wen TX Huang JL. Automated Alzheimer's disease classification using deep learning models with soft-Nms and improved Resnet50 integration. J Radiat Res Appl Sci. (2024) 17:100782. doi: 10.1016/j.jrras.2023.100782, PMID: 40630313

[26] El-Assy AM Amer HM Ibrahim HM Mohamed MA. A novel CNN architecture for accurate early detection and classification of Alzheimer's disease using MRI data. Sci Rep. (2024) 14:19. doi: 10.1038/s41598-024-53733-6 38342924 PMC10859371

[27] Rukhsar S Tiwari AK. Lightweight convolution transformer for cross-patient seizure detection in multi-channel EEG signals. Comput Methods Prog Biomed. (2023) 242:10. doi: 10.1016/j.cmpb.2023.107856, PMID: 37857026

[28] Li Z Fields M Panov F Ghatan S Yener B Marcuse L. Deep learning of simultaneous intracranial and scalp EEG for prediction, detection, and lateralization of mesial temporal lobe seizures. Front Neurol. (2021) 12:10. doi: 10.3389/fneur.2021.705119 PMC8632629 34867707

[29] Jusseaume K Valova I. Brain age prediction/classification through recurrent deep learning with electroencephalogram recordings of seizure subjects. Sensors. (2022) 22:28. doi: 10.3390/s22218112, PMID: 36365809 PMC9655329

[30] Soylu E Guel S Koca KA Tuerkoglu M Terzi M Senguer A. Speech signal-based accurate neurological disorders detection using convolutional neural network and recurrent neural network based deep network. Eng Appl Artif Intell. (2025) 149:15. doi: 10.1016/j.engappai.2025.110558

[31] Wang MK Hou SJ Wei Y Li DM Lin JP. Discovery of novel dual adenosine A1/A2a receptor antagonists using deep learning, pharmacophore modeling and molecular docking. PLoS Comput Biol. (2021) 17:e1008821. doi: 10.1371/journal.pcbi.1008821, PMID: 33739970 PMC7978378

[32] Zhang XL Che C. Drug repurposing for Parkinson's disease by integrating knowledge graph completion model and knowledge fusion of medical literature. Fut Internet. (2021) 13:13. doi: 10.3390/fi13010014

[33] Wang SD Du ZZ Ding M Rodriguez-Paton A Song T. Kg-dti: a knowledge graph based deep learning method for drug-target interaction predictions and Alzheimer's disease drug repositions. Appl Intell. (2022) 52:846–57. doi: 10.1007/s10489-021-02454-8

[34] Gao ZX Ding PJ Xu R. Kg-predict: a knowledge graph computational framework for drug repurposing. J Biomed Inform. (2022) 132:9. doi: 10.1016/j.jbi.2022.104133 PMC9595135 35840060

[35] Lin JZ He YJ Ru CX Long WL Li ML Wen ZN. Advancing adverse drug reaction prediction with deep chemical language model for drug safety evaluation. Int J Mol Sci. (2024) 25:13. doi: 10.3390/ijms25084516 PMC11050562 38674100

[36] Sun R Zhang WB Bagic A He B. Deep learning based source imaging provides strong sublobar localization of epileptogenic zone from meg Interictal spikes. NeuroImage. (2023) 281:13. doi: 10.1016/j.neuroimage.2023.120366, PMID: 37716593 PMC10771628

[37] Raymond C Zhang D Cabello J Liu LS Moyaert P Burneo JG. Smart-pet: a self-similarity-aware generative adversarial framework for reconstructing low-count 18f -fdg-pet brain imaging. Front Nucl Med. (2024) 4:1469490. doi: 10.3389/fnume.2024.1469490 39628873 PMC11611550

[38] Zhang JH Zhang XL Sh Y Liu BL Hu ZY. Diagnostic AI modeling and pseudo time series profiling of ad and pd based on individualized serum proteome data. Front Bioinformatics. (2021) 1:764497. doi: 10.3389/fbinf.2021.764497 PMC9581001 36303784

[39] Bayram B Kunduracioglu I Ince S Pacal I. A systematic review of deep learning in Mri-based cerebral vascular occlusion-based brain diseases. Neuroscience. (2025) 568:76–94. doi: 10.1016/j.neuroscience.2025.01.020, PMID: 39805420

[40] Soman K Nelson CA Cerono G Goldman SM Baranzini SE Brown EG. Early detection of Parkinson's disease through enriching the electronic health record using a biomedical knowledge graph. Front Med. (2023) 10:11. doi: 10.3389/fmed.2023.1081087 PMC10217780 37250641

[41] Yang JJ Gessner CR Duerksen JL Biber D Binder JL Ozturk M. Knowledge graph analytics platform with Lincs and Idg for Parkinson's disease target illumination. BMC Bioinformatics. (2022) 23:15. doi: 10.1186/s12859-021-04530-9 35021991 PMC8756622

[42] Yang SJ Chen SY Huang YL Lu Y Chen Y Ye LY. Combining MRI radiomics and clinical features for early identification of drug-resistant epilepsy in people with newly diagnosed epilepsy. Epilepsy Behav. (2025) 162:110165. doi: 10.1016/j.yebeh.2024.110165 39612633

[43] Wang YP Dai Y Liu ZM Guo JJ Cao GP Ouyang MW. Computer-aided intracranial Eeg signal identification method based on a multi-branch deep learning fusion model and clinical validation. Brain Sci. (2021) 11:23. doi: 10.3390/brainsci11050615, PMID: 34064889 PMC8150766

[44] Wang YP Yang YF Cao GP Guo JJ Wei PH Feng T. Seeg-net: an explainable and deep learning-based cross-subject pathological activity detection method for drug-resistant epilepsy. Comput Biol Med. (2022) 148:15. doi: 10.1016/j.compbiomed.2022.105703 35791972

[45] Boniolo F Dorigatti E Ohnmacht AJ Saur D Schubert B Menden MP. Artificial intelligence in early drug discovery enabling precision medicine. Expert Opin Drug Discov. (2021) 16:991–1007. doi: 10.1080/17460441.2021.1918096, PMID: 34075855

[46] Murali V Muralidhar YP Königs C Nair M Madhu S Nedungadi P. Predicting clinical trial outcomes using drug bioactivities through graph database integration and machine learning. Chem Biol Drug Des. (2022) 100:169–84. doi: 10.1111/cbdd.14092, PMID: 35587730

[47] Yang M Zhao YZ Yu HH Chen SL Gao GS Li D. A multi-label deep learning model for detailed classification of Alzheimer's disease. Actas Esp Psiquiatr. (2025) 53:89–99. doi: 10.62641/aep.v53i1.1728, PMID: 39801412 PMC11726212

[48] D'Sa K Evans JR Virdi GS Vecchi G Adam A Bertolli O. Prediction of mechanistic subtypes of Parkinson's using patient-derived stem cell models. Nat Mach Intell. (2023) 5:933–46. doi: 10.1038/s42256-023-00702-9, PMID: 37615030 PMC10442231

[49] Khatri U Kwon GR. Explainable vision transformer with self-supervised learning to predict Alzheimer's disease progression using 18f-Fdg pet. Bioengineering-Basel. (2023) 10:20. doi: 10.3390/bioengineering10101225, PMID: 37892955 PMC10603890

[50] Schuhholz M Ruff C Bürkle E Feiweier T Clifford B Kowarik M. Ultrafast brain MRI at 3 T for Ms: evaluation of a 51-second deep learning-enhanced T2-epi-flair sequence. Diagnostics. (2024) 14:33. doi: 10.3390/diagnostics14171841 PMC11393910 39272626

[51] Yang LQ Wang XF Guo Q Gladstein S Wooten D Li TF. Deep learning based multimodal progression modeling for Alzheimer's disease. Stat Biopharm Res. (2021) 13:337–43. doi: 10.1080/19466315.2021.1884129

[52] Niyas S Vaisali SC Show I Chandrika TG Vinayagamani S Kesavadas C. Segmentation of focal cortical dysplasia lesions from magnetic resonance images using 3d convolutional neural networks. Biomed Signal Process Control. (2021) 70:11. doi: 10.1016/j.bspc.2021.102951

[53] Alshaya H Hussain M. Eeg-based classification of epileptic seizure types using deep network model. Mathematics. (2023) 11:28. doi: 10.3390/math11102286, PMID: 40607831

[54] West M Cheng Y He YN Leng Y Magdamo C Hyman B. Unsupervised deep learning of electronic health records to characterize heterogeneity across Alzheimer disease and related dementias: cross-sectional study. JMIR Aging. (2025) 8:e65178. doi: 10.2196/65178 40163031 PMC11997524

[55] Shin DH Heo H Song S Shin NY Nam Y Yoo SW. Automated assessment of the substantia Nigra on susceptibility map-weighted imaging using deep convolutional neural networks for diagnosis of idiopathic Parkinson's disease. Parkinsonism Relat Disord. (2021) 85:84–90. doi: 10.1016/j.parkreldis.2021.03.004, PMID: 33761389

[56] Shen MT Mortezaagha P Rahgozar A. Explainable artificial intelligence to diagnose early Parkinson's disease via voice analysis. Sci Rep. (2025) 15:19. doi: 10.1038/s41598-025-96575-6 40188263 PMC11972358

[57] Yang SJ Xue JQ Li ZQ Zhang SQ Zhang Z Huang ZF. Deep learning-based ion channel kinetics analysis for automated patch clamp recording. Adv Sci. (2025) 12:17. doi: 10.1002/advs.202404166 PMC12083860 39737527

[58] Ren HH Song HJ Cui SG Xiong H Long BY Li YM. Deep learning of noncontrast CT for fast prediction of hemorrhagic transformation of acute ischemic stroke: a multicenter study. Eur Radiol Exp. (2025) 9:11. doi: 10.1186/s41747-024-00535-0 39812734 PMC11735721

[59] Sharma R Anand H Badr Y Qiu RG. Time-to-event prediction using survival analysis methods for Alzheimer's disease progression. Alzheimer’s Dement. (2021) 7:11. doi: 10.1002/trc2.12229, PMID: 35005207 PMC8719343

[60] Jeong JW Lee MH Kuroda N Sakakura K O'Hara N Juhasz C. Multi-scale deep learning of clinically acquired multi-modal Mri improves the localization of seizure onset zone in children with drug-resistant epilepsy. IEEE J Biomed Health Inform. (2022) 26:5529–39. doi: 10.1109/jbhi.2022.3196330, PMID: 35925854 PMC9710730

[61] Peterson V Kokkinos V Ferrante E Walton A Merk T Hadanny A. Deep net detection and onset prediction of electrographic seizure patterns in responsive Neurostimulation. Epilepsia. (2023) 64:2056–69. doi: 10.1111/epi.17666, PMID: 37243362

[62] Nadarajah R Wu JH Frangi AF Hogg D Cowan C Gale C. Predicting patient-level new-onset atrial fibrillation from population-based nationwide electronic health records: protocol of find-af for developing a precision medicine prediction model using artificial intelligence. BMJ Open. (2021) 11:7. doi: 10.1136/bmjopen-2021-052887 PMC8565546 34728455

[63] Pan YT Park K Ren JX Volkow ND Ling HB Koretsky AP. Dynamic 3d imaging of cerebral blood flow in awake mice using self-supervised-learning-enhanced optical coherence Doppler tomography. Commun Biol. (2023) 6:14. doi: 10.1038/s42003-023-04656-x, PMID: 36944712 PMC10030663

[64] Abousaber I. A novel explainable attention-based meta-learning framework for imbalanced brain stroke prediction. Sensors (Basel). (2025) 25:32. doi: 10.3390/s25061739 PMC11945820 40292890

[65] Galarza J Oraby T. Functional data learning using convolutional neural networks. Mach Learn-Sci Technol. (2024) 5:38. doi: 10.1088/2632-2153/ad2627

[66] Zhou C Cai CP Huang XT Wu S Yu JL Wu JW. Tarkg: a comprehensive biomedical knowledge graph for target discovery. Bioinformatics. (2024) 40:10. doi: 10.1093/bioinformatics/btae598, PMID: 39392404 PMC11513019

[67] Romano JD Truong V Kumar R Venkatesan M Graham BE Hao Y. The Alzheimer's Knowledge Base: a knowledge graph for Alzheimer disease research. J Med Internet Res. (2024) 26:e46777. doi: 10.2196/46777, PMID: 38635981 PMC11066745

[68] Zheng SJ Rao JH Song Y Zhang JX Xiao XL Fang EF. Pharmkg: a dedicated knowledge graph benchmark for Bomedical data mining. Brief Bioinform. (2021) 22:15. doi: 10.1093/bib/bbaa344, PMID: 33341877

[69] Nian Y Hu XY Zhang R Feng JN Du JC Li F. Mining on Alzheimer's diseases related knowledge graph to identity potential ad-related semantic triples for drug repurposing. BMC Bioinformatics. (2022) 23:15. doi: 10.1186/s12859-022-04934-1, PMID: 36180861 PMC9523633

[70] Kastrin A Hristovski D. Scientometric analysis and knowledge mapping of literature-based discovery (1986-2020). Scientometrics. (2021) 126:1415–51. doi: 10.1007/s11192-020-03811-z

[71] Singha M Pu LM Stanfield BA Uche IK Rider PJF Kousoulas KG. Artificial intelligence to guide precision anticancer therapy with multitargeted kinase inhibitors. BMC Cancer. (2022) 22:17. doi: 10.1186/s12885-022-10293-0 36434556 PMC9694576

[72] Gnanadesigan NS Dhanasegar N Ramasamy MD Muthusamy S Mishra OP Pugalendhi GK. An integrated network topology and deep learning model for prediction of Alzheimer disease candidate genes. Soft Comput. (2023) 27:14189–203. doi: 10.1007/s00500-023-08390-8

[73] Koirala S Samanta S Kar P. Identification of inhibitors for neurodegenerative diseases targeting dual leucine zipper kinase through virtual screening and molecular dynamics simulations. SAR QSAR Environ Res. (2024) 35:457–82. doi: 10.1080/1062936x.2024.2363195, PMID: 38855951

[74] Yao CP Shen ZY Shen LT Kadier K Zhao JY Guo Y. Identification of potential Jnk3 inhibitors: a combined approach using molecular docking and deep learning-based virtual screening. Pharmaceuticals. (2023) 16:13. doi: 10.3390/ph16101459 PMC10610115 37895928

[75] Raschka T Sood M Schultz B Altay A Ebeling C Fröhlich H. Ai reveals insights into link between Cd33 and cognitive impairment in Alzheimer's disease. PLoS Comput Biol. (2023) 19:e1009894. doi: 10.1371/journal.pcbi.1009894, PMID: 36780558 PMC9956604

[76] Neelakandan AR Rajanikant GK. A deep learning and docking simulation-based virtual screening strategy enables the rapid identification of Hif-1α pathway activators from a marine natural product database. J Biomol Struct Dyn. (2024) 42:629–51. doi: 10.1080/07391102.2023.2194997 37038705

[77] Yang LJ Yang GH Chen XL Yang Q Yao XJ Bing ZT. Deep scoring neural network replacing the scoring function components to improve the performance of structure-based molecular docking. ACS Chem Neurosci. (2021) 12:2133–42. doi: 10.1021/acschemneuro.1c00110, PMID: 34081851

[78] Ozalp MK Vignaux PA Puhl AC Lane TR Urbina F Ekins S. Sequential contrastive and deep learning models to identify selective Butyrylcholinesterase inhibitors. J Chem Inf Model. (2024) 64:3161–72. doi: 10.1021/acs.jcim.4c00397, PMID: 38532612 PMC11331448

[79] Gou RP Yang JY Guo MH Chen YJ Xue WW. Cnsmolgen: a bidirectional recurrent neural network-based generative model for De novo central nervous system drug design. J Chem Inf Model. (2024) 64:4059–70. doi: 10.1021/acs.jcim.4c00504, PMID: 38739718

[80] Liu DY Song T Na K Wang SD. Ped: a novel predictor-encoder-decoder model for Alzheimer drug molecular generation. Front Artif Intell. (2024) 7:1374148. doi: 10.3389/frai.2024.1374148 38690194 PMC11058643

[81] Jing YK Zhao GY Xu YY McGuire T Hou GQ Zhao JC. Gcn-Bbb: deep learning blood-brain barrier (Bbb) permeability pharmacoanalytics with graph convolutional neural (Gcn) network. AAPS J. (2025) 27:14. doi: 10.1208/s12248-025-01059-0 40180695

[82] Meng PR Mu WJ Ding DB Chen H Li ZH Hou HW. Discovery of positive allosteric modulators of Α7 nachr by an ensemble-based virtual screening method, molecular dynamics simulation, and *in vitro* biological activity testing. J Comput Biophys Chem. (2024) 23:925–37. doi: 10.1142/s2737416524500200

[83] Sutthibutpong T Posansee K Liangruksa M Termsaithong T Piyayotai S Phitsuwan P. Combining deep learning and structural modeling to identify potential acetylcholinesterase inhibitors from Hericium Erinaceus. ACS Omega. (2024) 9:16311–21. doi: 10.1021/acsomega.3c10459, PMID: 38617639 PMC11007777

[84] Wang H Xie MQ Rizzi G Li X Tan K Fussenegger M. Identification of sclareol as a natural neuroprotective Cav1.3-antagonist using synthetic Parkinson-mimetic gene circuits and computer-aided drug discovery. Adv Sci. (2022) 9:13. doi: 10.1002/advs.202102855 PMC8895113 35040584

[85] Mansingh P Pattanayak BK Pati B. Deep learning-based sentiment analysis for the prediction of Alzheimer's drugs. Comput Sist. (2023) 27:979–89. doi: 10.13053/CyS-27-4-4634

[86] Hakeem H Feng W Chen ZB Choong J Brodie MJ Fong SL. Development and validation of a deep learning model for predicting treatment response in patients with newly diagnosed epilepsy. JAMA Neurol. (2022) 79:986–96. doi: 10.1001/jamaneurol.2022.2514, PMID: 36036923 PMC9425285

[87] Cho D Yu MS Shin J Lee JY Kim Y Kang HC. A computational clinical decision-supporting system to suggest effective anti-epileptic drugs for pediatric epilepsy patients based on deep learning models using patient's medical history. BMC Med Inform Decis Mak. (2024) 24:9. doi: 10.1186/s12911-024-02552-w 38822293 PMC11143596

[88] Chang BW Geng Z Mei JM Wang ZY Chen P Jiang YG. Application of multimodal deep learning and multi-instance learning fusion techniques in predicting Stn-Dbs outcomes for Parkinson's disease patients. Neurotherapeutics. (2024) 21:9. doi: 10.1016/j.neurot.2024.e00471 PMC11585874 39419638

[89] Miranda O Jiang C Qi XG Kofler J Sweet RA Wang LR. Exploring potential medications for Alzheimer's disease with psychosis by integrating drug target information into deep learning models: a data-driven approach. Int J Mol Sci. (2025) 26:20. doi: 10.3390/ijms26041617, PMID: 40004081 PMC11855865

[90] Wui Y Liu Q Qiu Y Xie L. Deep learning prediction of chemical-induced dose-dependent and context-specific multiplex phenotype responses and its application to personalized Alzheimer's disease drug repurposing. PLoS Comput Biol. (2022) 18:28. doi: 10.1371/journal.pcbi.1010367 PMC9398009 35951653

[91] He S Abarrategi JS Bediaga H Arrasate S González-Díaz H. On the additive artificial intelligence-based discovery of nanoparticle neurodegenerative disease drug delivery systems. Beilstein J Nanotechnol. (2024) 15:535–55. doi: 10.3762/bjnano.15.47, PMID: 38774585 PMC11106676

[92] Kim H Park C Kim JH Jang S Lee HK. Multimodal reinforcement learning for embedding networks and medication recommendation in Parkinson's disease. IEEE Access. (2024) 12:74251–67. doi: 10.1109/access.2024.3405009

[93] Wu Z Yao T Wang Z Liu B Wu N Lu M. Association between angiotensin-converting enzyme inhibitors and the risk of lung Cancer: a systematic review and Meta-analysis. Br J Cancer. (2023) 128:168–76. doi: 10.1038/s41416-022-02029-5, PMID: 36396817 PMC9670057

[94] Dimoudis D Tsolakis N Magga-Nteve C Meditskos G Vrochidis S Kompatsiaris I. Inseption: a robust mechanism for predicting fog episodes in Pd patients. Electronics. (2023) 12:18. doi: 10.3390/electronics12092088

[95] Skampardoni I Nasrallah IM Abdulkadir A Wen JH Melhem R Mamourian E. Genetic and clinical correlates of Ai-based brain aging patterns in cognitively unimpaired individuals. JAMA Psychiatry. (2024) 81:456–67. doi: 10.1001/jamapsychiatry.2023.5599, PMID: 38353984 PMC10867779

[96] Mezzina G De Venuto D. A digital architecture for the real-time tracking of wearing off phenomenon in Parkinson's disease. Sensors. (2022) 22:15. doi: 10.3390/s22249753, PMID: 36560122 PMC9780967

[97] Sand D Rappel P Marmor O Bick AS Arkadir D Lu BL. Machine learning-based personalized subthalamic biomarkers predict on-off levodopa states in Parkinson patients. J Neural Eng. (2021) 18:17. doi: 10.1088/1741-2552/abfc1d, PMID: 33906182

[98] Fasano A Helmich RC. Tremor habituation to deep brain stimulation: underlying mechanisms and solutions. Mov Disord. (2019) 34:1761–73. doi: 10.1002/mds.27821, PMID: 31433906

[99] Oliveira A Coelho L Carvalho E Ferreira-Pinto MJ Vaz R Aguiar P. Machine learning for adaptive deep brain stimulation in Parkinson’s disease: closing the loop. J Neurol. (2023) 270:5313–26. doi: 10.1007/s00415-023-11873-1, PMID: 37530789 PMC10576725

[100] Zhong Y Wang Y He Z Lin Z Pang N Niu L. Closed-loop wearable ultrasound deep brain stimulation system based on Eeg in mice. J Neural Eng. (2021) 18:0460e8. doi: 10.1088/1741-2552/ac1d5c, PMID: 34388739

[101] Uchitel J Vidal-Rosas EE Cooper RJ Zhao H. Wearable, integrated EEG–FNIRS technologies: a review. Sensors (Basel). (2021) 21:6106. doi: 10.3390/s21186106, PMID: 34577313 PMC8469799

[102] Yang Y Truong ND Eshraghian JK Nikpour A Kavehei O. Weak self-supervised learning for seizure forecasting: a feasibility study. R Soc Open Sci. (2022) 9:220374. doi: 10.1098/rsos.220374, PMID: 35950196 PMC9346358

[103] Zhang H Chen Y Xie Y Chai Y. Closed-loop controller based on reference signal tracking for absence seizures. Sci Rep. (2022) 12:6730. doi: 10.1038/s41598-022-10803-x, PMID: 35468988 PMC9038751

[104] Farronato M Mannocci P Milozzi A Compagnoni CM Barcellona A Arena A. Seizure detection via reservoir computing in MoS(2)-based charge trap memory devices. Sci Adv. (2025) 11:eadr3241. doi: 10.1126/sciadv.adr3241, PMID: 39823342 PMC11740968

[105] Ronchini M Rezaeiyan Y Zamani M Panuccio G Moradi F. Net-ten: a silicon neuromorphic network for low-latency detection of seizures in local field potentials. J Neural Eng. (2023) 20:036002. doi: 10.1088/1741-2552/acd029, PMID: 37144338

[106] Zhou A Santacruz SR Johnson BC Alexandrov G Moin A Burghardt F. A wireless and artefact-free 128-channel neuromodulation device for closed-loop stimulation and recording in non-human primates. Nat Biomed Eng. (2018) 3:15–26. doi: 10.1038/s41551-018-0323-x, PMID: 30932068

[107] Baghersalimi S Teijeiro T Atienza D Aminifar A. Personalized real-time federated learning for epileptic seizure detection. IEEE J Biomed Health Inform. (2022) 26:898–909. doi: 10.1109/jbhi.2021.3096127, PMID: 34242177

[108] Ganji Z Azizi S Faraji R Zare H. Application of neuroimaging in diagnosis of focal cortical dysplasia: a survey of computational techniques. Neurocomputing. (2024) 580:127418. doi: 10.1016/j.neucom.2024.127418, PMID: 40630313

[109] Xiao YK Zhang SN Zhou HX Li MC Yang H Zhang R. Fuselinker: leveraging LLM'S ' s pre-trained text embeddings and domain knowledge to enhance GNN-based link prediction on biomedical knowledge graphs. J Biomed Inform. (2024) 158:104730. doi: 10.1016/j.jbi.2024.104730 39326691 PMC12079804

[110] Massoud YM Ahmad AA Abdelzaher M Kuhlmann L Abd El Ghany MA. Hardware implementation of deep neural network for seizure prediction. AEU-Int J Electron Commun. (2023) 172:154961. doi: 10.1016/j.aeue.2023.154961, PMID: 40630313

[111] Vatansever S Schlessinger A Wacker D Kanıskan HÜ Jin J Zhou MM. Artificial intelligence and machine learning-aided drug discovery in central nervous system diseases: state-of-the-arts and future directions. Med Res Rev. (2020) 41:1427–73. doi: 10.1002/med.21764, PMID: 33295676 PMC8043990

[112] Ponnapalli SP Miron P Miskimen K Waite K Sosonkina N Coppens SE. Abstract A031: prospective and clinical prediction in a retrospective trial that experimentally validated an AI/ML-derived whole-genome predictor as the most accurate and precise predictor of survival and response to treatment in glioblastoma. Cancer Res. (2024) 84:A031–A. doi: 10.1158/1538-7445.brain23-a031

[113] Piraino F Costa M Meyer M Cornish GH Ceroni C Garnier V. Organoid models: the future companions of personalized drug development. Biofabrication. (2024) 16:032009. doi: 10.1088/1758-5090/ad3e30, PMID: 38608454

[114] Marzi SJ Schilder BM Nott A Frigerio CS Willaime-Morawek S Bucholc M. Artificial Intelligence for Neurodegenerative Experimental Models. Alzheimer’s Dement. (2023) 19:5970–87. doi: 10.1002/alz.13479, PMID: 37768001

[115] Mittermaier M Raza M Kvedar JC. Bias in Ai-based models for medical applications: challenges and mitigation strategies. NPJ Digit Med. (2023) 6:113. doi: 10.1038/s41746-023-00858-z, PMID: 37311802 PMC10264403

[116] Vokinger KN Feuerriegel S Kesselheim AS. Continual learning in medical devices: Fda's action plan and beyond. Lancet Digit Health. (2021) 3:e337–8. doi: 10.1016/s2589-7500(21)00076-5, PMID: 33933404

[117] Vokinger KN Gasser U. Regulating Ai in medicine in the United States and Europe. Nat Mach Intell. (2021) 3:738–9. doi: 10.1038/s42256-021-00386-z, PMID: 34604702 PMC7611759

[118] Abualrob MA Itbaisha A Mesraoua B. Unlocking new frontiers in epilepsy through AI: from seizure prediction to personalized medicine. Epilepsy Behav. (2025) 166:8. doi: 10.1016/j.yebeh.2025.110327 40043598

[119] Price WN 2nd Cohen IG. Privacy in the age of medical big data. Nat Med. (2019) 25:37–43. doi: 10.1038/s41591-018-0272-7, PMID: 30617331 PMC6376961

[120] Wu Z Yao T Shen N. To face Disease X: building resilient futures in the age of emergent threats. BMJ Glob Health. (2025) 10:e020479. doi: 10.1136/bmjgh-2025-020479, PMID: 40617591 PMC12228457

